# A comprehensive risk map for malaria in Kinshasa, Democratic Republic of Congo

**DOI:** 10.1186/1475-2875-11-S1-O8

**Published:** 2012-10-15

**Authors:** Giovanfrancesco Ferrari, Henry Ntuku, Sandro Schmidlin, Christian Lengeler, Antoinette Tshefu

**Affiliations:** 1 Department of Epidemiology and Public Health, Swiss Tropical and Public Health Institute, Basel, Switzerland; 2 Ecole de Santé Publigue, Faculté de Médecine, The Democratic Republic of the Congo

## Background

The Democratic Republic of Congo (DRC) is the second most malarious country in the world. However, there is a paucity of epidemiological data on the risk pattern of malaria.

## Methods

In 2009 (dry season) and 2011 (end of the rainy season), two two-stage cluster sampling malaria surveys were conducted in the capital city Kinshasa with the twofold aim of (1) assessing malaria parasite prevalence, anemia and associated malaria risk factors, and (2) producing a malaria risk map using a geographic information system (GIS).

## Results

A total of 6410 children aged 6 - 59 months (3058 in 2009 and 3352 in 2011) were tested for both malaria (using rapid diagnostic tests) and anemia (by Hemocue™). Nine health zones (HZ) were sampled in 2009 with an average prevalence for malaria and anemia of 6.6% (95% CI 5.8 - 7.5) and 66.0% (64.5 - 67.4) respectively, while in the 25 HZs in 2011 the prevalence was 17.0% (15.7 - 18.3) and the anemia rate was 64.2% (62.6 - 65.9). Overall, the prevalence rate for both surveys was 11.9% (11.2 - 12.8) for malaria and 65.1% (63.9 - 66.7) for anemia. To ensure comparability of the results between surveys, two HZs from 2009 were resampled in 2011. Prevalence for malaria in 2009 and 2011 was:Ngiri Ngiri 1.0% versus 0.8% and Selembao: 14.1% versus 26.8%. Prevalence for anemia was: Ngiri Ngiri 62.5% versus 55.4% and Selembao: 67.1% versus 61.4%. In a multivariate analysis of the 2011 data, significant protective factors for malaria risk were: educational level of the respondent (OR = 0.12, 95% CI: 0.03 - 0.56) and sleeping under an ITN (OR = 0.52, 95% CI: 0.43 - 0.63). All key parameters were mapped to the level of the HZs (n = 35). Malaria parasitemia, anemia and fever prevalence were found to be much lower in the city center than in the peri-urban suburbs, where transmission rates remain high. ITN usage showed the opposite pattern.

## Conclusions

For the first time a comprehensive picture of the epidemiology of malaria has been prepared for Kinshasa, a mega-city in a highly endemic zone. This provides a solid baseline information for planning future malaria control interventions.

